# Characterization of BIS20x3, a bi-specific antibody activating and retargeting T-cells to CD20-positive B-cells

**DOI:** 10.1054/bjoc.2000.1707

**Published:** 2001-04

**Authors:** S Withoff, M N A Bijman, A J Stel, L Delahaye, A Calogero, M W A de Jonge, B J Kroesen, L de Leij

**Affiliations:** Department of Pathology and Laboratory Medicine, University Hospital Groningen, Section Medical Biology – Laboratory Tumor Immunology, Hanzeplein 1, GZ Groningen, 9713, The Netherlands; IQ Corporation, Zernikepark 6b, AN Groningen, 9747, The Netherlands

**Keywords:** BIS20x3, CD20, rituximab, bi-specific antibody, non-Hodgkin's lymphoma

## Abstract

This paper describes a bi-specific antibody, which was called BIS20x3. It retargets CD3ɛ-positive cells (T-cells) to CD20-positive cells and was obtained by hybrid–hybridoma fusion. BIS20x3 could be isolated readily from quadroma culture supernatant and retained all the signalling characteristics associated with both of its chains. Cross-linking of BIS20x3 on Ramos cells leads to DNA fragmentation percentages similar to those obtained after Rituximab-cross-linking. Cross-linking of BIS20x3 on T-cells using cross-linking F(ab′)2-fragments induced T-cell activation. Indirect cross-linking of T-cell-bound BIS20x3 via Ramos cells hyper-activated the T-cells. Furthermore, it was demonstrated that BIS20x3 effectively re-targets T-cells to B-cells, leading to high B-cell cytotoxicity. The results presented in this paper show that BIS20x3 is fully functional in retargeting T-cells to B-cells and suggest that B-cell lymphomas may represent ideal targets for T-cell retargeting bi-specific antibodies, because the retargeted T-cell is maximally stimulated in the presence of B-cells. Additionally, since B-cells may up-regulate CD95/ *Fas* expression upon binding of CD20-directed antibodies, B-cells will become even more sensitive for T-cell mediated killing via CD95L/ *Fas* L, and therefore supports the intention to use T-cell retargeting bi-specific antibodies recognizing CD20 on B-cell malignancies as a treatment modality for these diseases. © 2001 Cancer Research Campaign http://www.bjcancer.com


*British Journal of Cancer* (2001) **84**(8), 1115–1121
© 2001 Cancer Research Campaign
doi: 10.1054/ bjoc.2001.1707, available online at http://www.idealibrary.com on IDEAL®

### Characterization of BIS20x3, a bi-specific antibody activating and retargeting T-cells to CD20-positive B-cells

**S Withoff[1], MNA Bijman[1], AJ Stel[1], L Delahaye[1], A Calogero[1], MWA de Jonge[2], BJ Kroesen[1] and L de Leij[1]**

[1]University Hospital Groningen, Department of Pathology and Laboratory Medicine, Section Medical Biology – Laboratory Tumor Immunology, Hanzeplein 1, 9713 GZ Groningen, The Netherlands; [2]IQ Corporation, Zernikepark 6b, 9747 AN Groningen, The Netherlands

**Summary** This paper describes a bi-specific antibody, which was called BIS20x3. It retargets CD3ε-positive cells (T-cells) to CD20-positive cells and was obtained by hybrid–hybridoma fusion. BIS20x3 could be isolated readily from quadroma culture supernatant and retained all the signalling characteristics associated with both of its chains. Cross-linking of BIS20x3 on Ramos cells leads to DNA fragmentation percentages similar to those obtained after Rituximab-cross-linking. Cross-linking of BIS20x3 on T-cells using cross-linking F(ab′)2-fragments induced T-cell activation. Indirect cross-linking of T-cell-bound BIS20x3 via Ramos cells hyper-activated the T-cells. Furthermore, it was demonstrated that BIS20x3 effectively re-targets T-cells to B-cells, leading to high B-cell cytotoxicity. The results presented in this paper show that BIS20x3 is fully functional in retargeting T-cells to B-cells and suggest that B-cell lymphomas may represent ideal targets for T-cell retargeting bi-specific antibodies, because the retargeted T-cell is maximally stimulated in the presence of B-cells. Additionally, since B-cells may up-regulate CD95/*Fas* expression upon binding of CD20-directed antibodies, B-cells will become even more sensitive for T-cell mediated killing via CD95L/*Fas*L, and therefore supports the intention to use T-cell retargeting bi-specific antibodies recognizing CD20 on B-cell malignancies as a treatment modality for these diseases. © 2001 Cancer Research Campaign http://www.bjcancer.com

**Keywords**: BIS20x3; CD20; rituximab; bi-specific antibody; non-Hodgkin's lymphoma

The concept of using antibodies as 'magic bullets' for cancer therapy has shown a remarkable revival. Recently highly encouraging results have been reported with antibodies targeting EGP-2/EpCAM on carcinomas (Edrecolomab; Riethmuller et al, 1998), HER-2/*neu* on breast cancers (Trastuzumab; Cragg et al, 1999) and CD20 on lymphomas (Rituximab; Rosen, 1999). Impressive response rates are obtained with these antibodies alone or in combination with additional chemo-therapeutic and/or radio-therapeutic regimens, without showing significant cytotoxicity.

The most promising of these antibodies are those targeting the B-cell differentiation marker CD20. These antibodies have been used for treatment of non-Hodgkin lymphoma (NHL) patients, as more than 90% of all non-Hodgkin lymphomas are positive for CD20. After Rituximab-treatment 50% of patients with advanced, indolent NHL showed partial or complete responses (Rosen, 1999). Because CD20 is expressed on B-cells during a number of stages of B-cell development, but is absent from B-cell progenitor cells and mature plasma cells (Rudin and Thompson, 1998), anti-CD20-antibodies do compromise the patient's immune system only to a limited extent.

The effectivity of Rituximab can probably be explained by its supposed dual effector function. First it is known that antibodies retarget the immune system to antibody-coated cells via their Fc-tail, leading ultimately to death of the cell by complement mediated lysis and/or antibody-dependent cellular cytotoxicity. Secondly, it has been shown that binding of antibodies to CD20 can modulate B-cell proliferation and/or antibody-dependent cellular cytotoxicity and that cross-linking of anti-CD20-antibodies (e.g. by Fc-receptor positive cells) on B-cells can lead to apoptosis of the target cell (Shan et al, 1998, 2000). The latter effects are thought to contribute significantly to the efficiency of treatment with this 'signalling antibody' (Cragg et al, 1999).

Although the results obtained with Rituximab are very promising it is important to note that anti-CD20-treatment is not curative and that treated patients may relapse ultimately. In order to improve upon the efficiency of anti-CD20-antibody treatment several strategies have been investigated. One approach is the labelling of the antibodies with radioisotopes to induce local irradiation. In another approach toxins are coupled to the antibodies and by this means delivered to tumour cells. Also combination therapy regimens consisting of Rituximab and chemotherapy have been tested in clinical trials (Rosen, 1999). Although these approaches are indeed more successful (higher response rates were achieved) increased toxicity to normal tissues may be expected.

We have focused in the past on the development of bispecific antibodies which are designed to retarget T-cells to tumour (associated) antigens. In the setting of NHL it might be expected that bi-specific antibody-mediated retargeting of T-cells to CD20-positive tumour cells will induce apoptosis more efficiently than CD20-cross-linking alone, because T-cells are equipped to optimally induce apoptosis in target cells.

In this paper we describe the production and characterization of the bi-specific antibody BIS20x3 via hybrid hybridoma techniques. BIS20x3 recognizes CD20 with one chain and CD3ε

*Received 5 September 2000*
*Accepted 2 January 2001*

*Correspondence to:* L de Leij

(on T-cells) with the other. A highly efficient activation of T-cells was observed after application of BIS20x3 and subsequent indirect cross-linking via CD20-positive cells. The results presented in this paper demonstrate that BIS20x3 is an attractive candidate for being tested as a new treatment modality for CD20-positive malignancies. B-cell malignancies may be ideal targets for T-cell retargeting bi-specific antibodies because B-cells may efficiently co-stimulate the effector T-cells.

### MATERIALS AND METHODS

### Culture media and other reagents

RPMI 1640 medium (containing 25 mM Hepes and L-glutamin) and 100x HT-supplement were obtained from Bio-Whittaker (Verviers, Belgium) and FCS from Bodinco BV (Alkmaar, The Netherlands). Culturing 'additives' consist of 1 mM sodium pyruvate (Bio-Whittaker), 2 mM L-glutamin (Bio-Whittaker), 0.5 mM β-mercaptoethanol, 0.1 mg $ml^{-1}$ gentamycin sulphate (Bio-Whittaker), and fungizone (Amfotericine-B, Bristol-Myers Squibb, Woerden, The Netherlands). Hygromycin was purchased from Roche Molecular Biochemicals (Almere, The Netherlands), PFHM-II (protein-free hybridoma medium) containing glutamax-I from Gibco BRL-Life Technologies (Breda, The Netherlands), calcein-AM from Molecular Probes (Leiden, The Netherlands), protein-A (PROSEP®-A) from Bioprocessing Ltd (ImmunoSource, Zoersel-Halle, Belgium), and Triton X-100, propidium iodide (PI) and RNase-A from Sigma-Aldrich Chemie BV (Zwijndrecht, The Netherlands). Lymfoprep was obtained from Nycomed Pharma AS (Oslo, Norway) and serum- and phenolred-free medium (X-VIVO™) from Bio-Whittaker.

### Hybridomas, quadroma and cell lines

Hybridoma and quadroma cell lines were cultured in RPMI 1640 medium containing 15% FCS, 15% ESG (conditioned ES-1-culture medium, IQ-Products, Groningen, The Netherlands), additives (see above), and HT-supplement. All other cell lines were cultured in essentially the same medium, however without conditioned ES-1 culture medium and HT supplement. All cells were cultured at 37˚C, in a humidified atmosphere containing 5% $CO_2$. The EBV-immortalized human B-cell line JY was obtained from Dr L Bakker (Dept. Tumor Immunology, Nijmegen, the Netherlands). CTLL/CD3ε is a mouse T-cell line transfected with cDNA encoding human CD3ε (Helfrich et al, 1998). Jurkat-AM, which was obtained from Dr P Schrier (University Hospital Leiden, Leiden, The Netherlands), was cultured in the presence of 0.5 mg $ml^{-1}$ hygromycin in order to select for NFAT-luc (the reporter gene luciferase under control of the NFAT-promoter) positive cells.

### Commercial antibodies

FITC-, TRITC- or PE-labelled anti-lgG1- or anti-lgG2b-antibodies were obtained from Becton Dickinson (Erembodegem-Aalst, Belgium) or from IQ-Products (Groningen, the Netherlands). Goat-anti-mouse-F (ab′)2-fragments (GaM-F(ab′)2) and goat-anti-human-F(ab′)2-fragments (GaH-F(ab′)2) used for cross-linking of respectively mouse-and human anti-CD20-antibodies were obtained from Jackson Laboratories (West Grove, PA, USA) as well as anti-human lgM-antibody. The anti-CD3-antibody B-B11 was obtained from lQ-Products.

### Hybrid hybridoma fusion, subcloning of quadroma cells and characterization of the antibodies produced by the sub-clones

A quadroma cell line was derived after fusion of the hybridoma cell lines B-ly1 (anti-CD20, lgG1; Poppema and Visser, 1987) and CLB-T3/4.2B (anti-CD3ε, lgG2b; van Lier et al, 1987a,b). Fusion was performed by standard hybrid hybridoma fusion techniques as described previously (Kroesen et al, 1993). Briefly, a HGPRT-negative anti-CD3ε hybridoma cell line was selected by culturing it in 8-azaguanine containing medium. This hybridoma cell line, unable to grow in hypoxantine, aminopterine- and thymidine-(HAT) containing medium, was made neomycin resistant by retroviral transfection. This HAT sensitive, neomycin-resistant cell line was then fused with the HAT-resistant, neomycin-sensitive hybridoma cell line B-ly1. Standard polyethyleneglycol (PEG) fusion procedures were used. Quadroma cells were subcloned in 96-well plates and clones producing both IgG1- and IgG2b-chains were identified by detection of fluorescence after labelling the quadroma cells with FITC- or TRITC-labelled anti-IgG1- or anti-IgG2b-antibodies. One subclone was selected. In order to validate CD20- and CD3ε-binding capacity of the antibodies produced by this clone, culture supernatant was harvested and applied to CD20-positive JY cells or CD3ε-positive CTLL/CD3ε cells.

### Partial purification of BIS20x3

Twice weekly antibody-producing cells were seeded in PFHM-II medium. Supernatant was harvested when the number of dead cells exceeded 30%. Approximately 500 ml supernatant was collected and concentrated 10× using the ProVario-3FP concentrator (Filtron Technology BV, Terheyden, the Netherlands). Antibody purification was performed by FPLC (Pharmacia, Uppsala, Sweden). Concentrated supernatant was diluted two-fold in 0.1 M K-phosphate buffer pH 8.2 (equilibration buffer) and loaded onto a protein-A column. Antibodies were eluted using a 0.1 M Na-citrate buffer pH-gradient, decreasing from pH 6 to pH 2. Supernatants of the parental hybridomas were subjected to the same procedure in order to determine at which pH the parental antibodies elute from the column. During elution protein peaks were detected spectrophotometrically (OD280) and 0.5 ml fractions were collected. In each fraction the pH was measured and neutralized by adding increasing volumes of a 1 M K-phosphate-solution (pH 9.4). Each fraction was analysed for the presence of IgG1- and/or IgG2b-isotype antibody chains by FACS analysis (Epics Elite, Coulter, Hialeah, USA) using the antibodies described above. The presence of the various antibody species was confirmed by SDS/PAGE gel electrophoresis and subsequent Western blotting.

### Detection of DNA fragmentation after cross-linking of anti-CD20-antibodies on CD20-positive cells

To induce apoptosis Ramos cells were harvested and incubated for 30 min with the different anti-CD20-antibody concentrations (see Results) at 37˚C. The cells were washed to remove unbound antibody and plated at a concentration of 0.5–1 x $10^6$ per well (6 well plates, 2.5 ml per well). Cross-linking was achieved by adding

*© 2001 Cancer Research Campaign*

cross- linking F(ab′)2-fragments. The next day the Nicoletti assay was performed to determine the percentage (%) apoptotic cells in the population. This assay is based on detection of a hypo-ploid DNA peak by FACS analysis (Nicoletti et al, 1991). After 16 h apoptosis induction the cells were washed in PBS containing 1% BSA and resuspended in 0.2% Na-citrate/0.2% Triton X-100/ 0.2 mg ml$^{-1}$ RNase-A. DNA was stained by adding 100 μg ml$^{-1}$ PI. PI fluorescence was measured by FACS.

### Detection of T-cell activation after CD3ε-cross-linking

A TCR-beta chain negative Jurkat cell line harbouring an NFAT-luciferase reporter gene named Jurkat-AM, was retrovirally transduced with genes encoding the alpha and beta TCR chains from an HLA-A2-restricted anti-MAGE-3 T-cell clone (van der Bruggen et al, 1994). The resulting subline is called Jurkat-AM/T. Transduction restored CD3ε-expression on the surface of Jurkat-AM/T as was determined by FACS (not shown). After binding of anti-CD3ε-antibodies to Jurkat-AM/T, the NFAT-promoter becomes activated which results in luciferase expression. Approximately 1.5 x $10^6$ cells per well (24 well plates) were activated during the period chosen. When necessary 0.5 x $10^6$ Ramos cells were added. The cells were harvested and washed once. Pellets were frozen at –20˚C for at least 15 min to facilitate lysis which was performed in lysis buffer supplied by the manufacturer of the Luciferase Assay System (Promega, Leiden, The Netherlands). Luciferase activity was detected using the same kit. Luminescence was detected on the Anthos Lucy Microplate Luminometer and Photometer (Labtech International, Ringmer, UK).

### Cytotoxicity assays

We have used the calcein-release assay (Lichtenfels et al, 1994) as an alternative for the $^{51}$Cr-release assay to demonstrate that BIS20x3 re-targets T-cells to CD20-positive cells. PBLs were isolated from heparin-blood using lymfoprep according to protocols described by the manufacturer. T-cells were activated and expanded by incubation for 3 days in RPMI 1640 medium containing 15% normal human poolserum and 5% WT-32 (anti-CD3ε-hybridoma) supernatant (Tax et al, 1983), followed by 2 days culturing in RPMI 1640 medium containing 15% normal human poolserum and 100 IU ml$^{-1}$ IL-2. JY-cells were used as the CD20-positive target cells. JY cells were isolated, washed and resuspended in X-VIVO-medium at a concentration of 2 x $10^6$ cells ml$^{-1}$. Calcein-AM was added to the cells in a concentration of 8 μM for 40 min, after which extracellular calcein-AM was removed by washing. Calcein-AM is the acetoxy-methylester of calcein which diffuses passively across cell membranes. In the cytosol calcein-AM is converted into the fluorochrome calcein by intracellular esterases. Calcein is retained in cells with intact membranes due to its polar nature. We applied 2 x $10^4$ labelled JY target cells per well to 96-well plates, and varied the number of T-cells (also washed and resuspended in X-VIVO-medium) to obtain varying effector-target cell ratios. After 2 h incubation in the absence or presence of different antibodies, the cells in the plates were pelleted and the supernatant was transferred into a new 96-well plate. Calcein fluorescence in the supernatant was determined on the Bio-Tek FL500 fluorescence plate reader (BioTek® Instruments Inc, Burlington, USA; excitation at 485 nm, emission at 530 nm). The % cytotoxicity was calculated using the equation: $(F_{sample} - F_{spontaneous\ release}) / (F_{total\ lysis} - F_{spontaneous\ release})$ x 100% = % cytotoxicity. Total lysis values were obtained by addition of 0.5% Triton X-100 to labelled JY cells. The $^{51}$Cr-release assay was performed as described previously (Kroesen et al, 1995).

### RESULTS

### Hybrid hybridoma fusion

The quadroma cell line producing BIS20x3 was made by fusion of B-ly1 and CLB-T3/4.2B. The quadroma was screened for expression of both parental antibody chains and cultured after subcloning twice. A clone displaying more than 95% double positive cells was selected as the 'producer' cell line. Additionally, culture supernatant was applied to CD20- or CD3ε-positive cell lines (JY or CTLL/CD3ε respectively). The co-presence of antibody-chain subclasses which do not bind by themselves on the target cells (anti-CD20-chains (of lgG1-subclass) on CTLL/CD3ε cells or anti-CD3ε-chains (of lgG2b-subclass) on JY-cells) suggested that the bi-specific antibody was formed and confirmed the antigen-binding capacity of both chains (results not shown).

### Isolation and partial purification of the bispecific antibody (BIS20x3)

Culture supernatants from B-ly1-(anti-CD20), CLB-T3/4.2B- (anti-CD3ε) or the quadroma culture were isolated and concentrated. First, samples of the concentrated parental hybridoma supernatants were mixed and loaded onto a protein-A FPLC column to elucidate the pH-elution profile of both parental antibodies. Using FACS- and Western blot analysis it was shown that the parental anti-CD20-antibody B-ly1 leaves the column between pH 5.5 and pH 4 and that the anti-CD3ε-antibody elutes between pH3 and 2 (peak at pH 2.5). In Figure 1 the elution profiles are depicted. The results suggest that the parental antibodies can be separated from each other. After loading quadroma supernatant and subsequent antibody elution using the same pH gradient, it was demonstrated that the bi-specific antibody leaves the column at a pH value lower than pH 3.8 indicating that the bi-specific antibody, which was called BIS20x3, could be isolated without 'contaminating' B-ly1. However, the BIS20x3 peak overlaps partially with the anti-CD3ε-peak. Therefore it was decided to use only the fractions isolated from the first half of the BIS20x3-peak for the experiments described below (more than 90% pure regarding BIS20x3-content). To check the

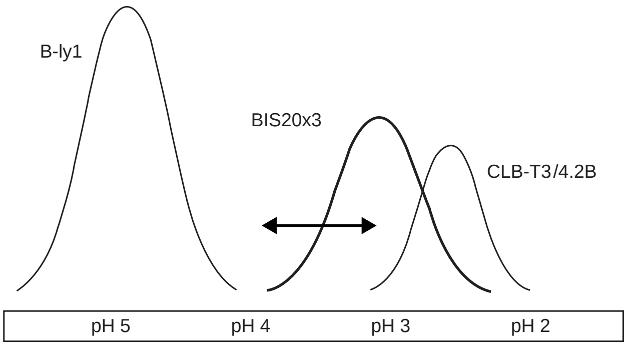

**Figure 1** Schematic representation of the pH elution profiles of both parental antibodies (B-ly1 and CLB-T3/4.2b) and the bispecific antibody (BIS20x3). The double arrow indicates the fractions used for the experiments described in this paper

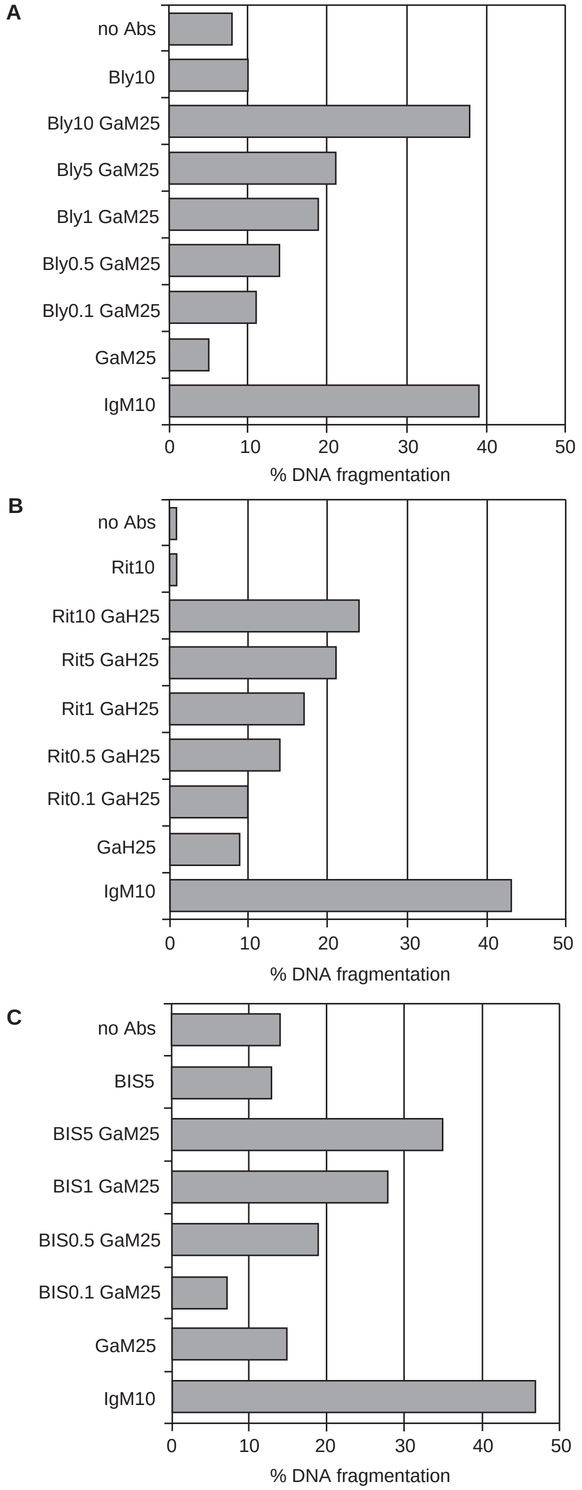

**Figure 2** Apoptosis induction in Ramos cells after cross-linking of anti-CD20 antibodies on the cell surface. In the experiment described in Figure 2A apoptosis was induced by cross-linking of B-ly1 which is the parental antibody used for derivation of BIS20x3. In Figure 2B results obtained after cross-linking of Rituximab are described. Figure 3C depicts results obtained after cross-linking of BIS20x3. Abbreviations used: no Abs = no antibodies, Bly = B-ly1, GaM = goat-anti-mouse-F(ab′)2-fragments, IgM = anti-IgM-antibody, Rit = Rituximab, GaH = goat-anti-human-F(ab′)2-fragments, Bis = BIS20x3. The numbers describe the amount of each antibody (or antibody-fragment) applied, in μg $ml^{-1}$. The data presented in this graph are representative for 3 or more experiments functionality of the antigen-binding capacity of both arms of the bispecific antibody, assays were subsequently performed which detect the intracellular signals which are relayed after binding of antibodies to CD20 on B-cells or CD3ε on T-cells, respectively.

### Detection of DNA hypo-ploidy after cross-linking of anti-CD20-antibodies on Ramos-cells

It was reported that binding of anti-CD20-antibodies to CD20 and subsequent cross-linking of these antibodies leads to apoptosis induction in Ramos cells (Shan et al, 1998). From Figure 2A and 2B it can be concluded that B-ly1 cross-linking leads to similar DNA-fragmentation percentages as when Rituximab is cross-linked. A dosage of only 0.1–0.5 μg $ml^{-1}$ B-ly1 or Rituximab is enough to detect apoptotic cells after cross-linking during 16 h. Also shown is that addition of anti-CD20 antibody alone or cross-linking antibody alone does not affect the Ramos cells. As a control anti-IgM-antibody was applied, resulting in high percentages of DNA fragmentation. Also cross-linking of BIS20x3 leads to DNA fragmentation in Ramos cells (Figure 2C), becoming evident at a BIS20x3 concentration of 0.5 μg $ml^{-1}$. The levels of DNA fragmentation appear to be higher than achieved in Figure 2A, B, however it has to be remarked that the untreated control shows already a relatively high apoptotic fraction in this experiment. The results presented here demonstrate that the anti-CD20 chain of BIS20x3 is functionally active in inducing apoptosis upon cross-linking.

### T-cell signalling induced by binding of BIS20x3 to Jurkat-AM/T cells

To demonstrate the functional capacity of the αCD3-chain of CD20x3 to activate T-cells, a CD3ε-signalling assay was used which was described in the Materials and methods section. Figure 3A shows results obtained after binding of BIS20x3 to CD3ε expressed on the surface of Jurkat-AM/T cells, and subsequent cross-linking of BIS20x3 by goat-anti-mouse-F(ab′)2-fragments. Cross-linking of increasing amounts of BIS20x3 results in increasing levels of T-cell stimulation. As a positive control T-cells were stimulated with WT-32 culture supernatant and as a negative control no antibody was added. Because we also expected indirect CD3ε-cross-linking to occur (and therefore T-cell activation) when Ramos cells were added to BIS20x3-coated Jurkat-AM/T cells, we performed the experiment summarized in Figure 3B. In this graph it is clearly shown that the T-cells are much more efficiently stimulated when BIS20x3 is cross-linked via Ramos cells than when the bi-specific antibody is cross-linked with goat-anti-mouse-F(ab′)2-fragments (Figure 3A), what may be caused by a co-stimulatory capacity of the Ramos cells. The results presented in Figure 3 demonstrate the functional binding of BIS20x3 to CD3ε.

### BIS20x3 re-targets PBL derived T-cells to CD20-positive JY cells

In Figure 4 representative results obtained with the calcein release assay are presented. In Figure 4A it is shown that when using the JY cell line, spontaneous release values were observed of approximately 25%. Neither the medium taken from unlabelled JY cell cultures nor medium from unlabelled T-cell cultures displayed

*© 2001 Cancer Research Campaign*

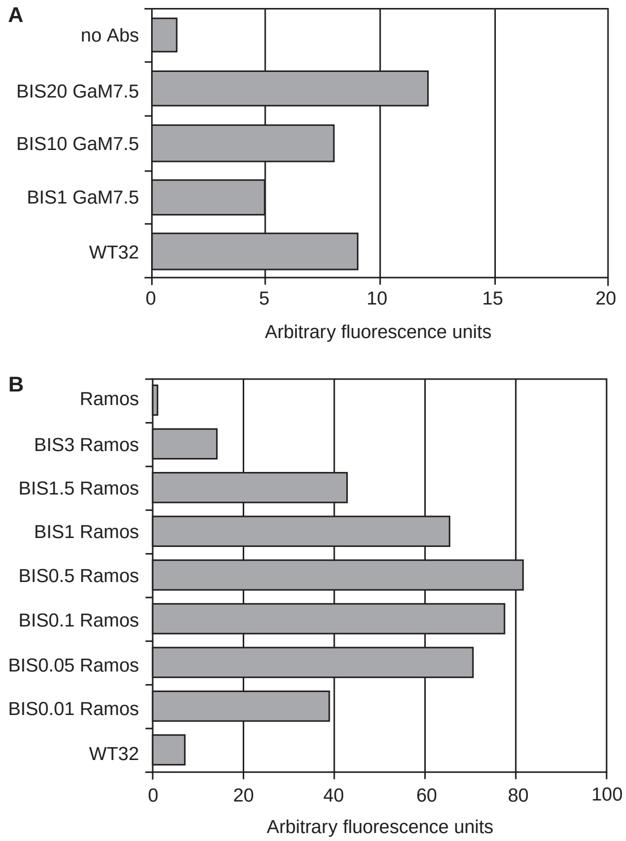

**Figure 3** Luciferase activity measured in extracts of Jurkat-AM/T cells after different treatments. In Figure 3A it is shown that cross-linking of increasing amounts of BIS20x3 (BIS) using goat-anti-mouse-F(ab′)2-fragments (GaM) leads to increasing T-cell activation. As a negative control no antibodies (no Abs) were added and as a positive control 100 µl WT-32 supernatant (WT32) was applied. In Figure 3B the remarkable results obtained after indirect cross-linking via Ramos cells are presented. Cross-linking of varying amounts (the numbers represent µg ml$^{-1}$) of BIS20x3 on the surface of Jurkat-AM/T-cells via Ramos cells, leads to a dramatic increase in T-cell activation when compared with the results obtained after cross-linking using goat-anti-mouse-F(ab′)2-fragments. At higher levels of BIS20x3 the amount of bispecific antibody saturates the available binding sites on both T- and B-cells which prevents cross-linking and results in a decrease in T-cell activation

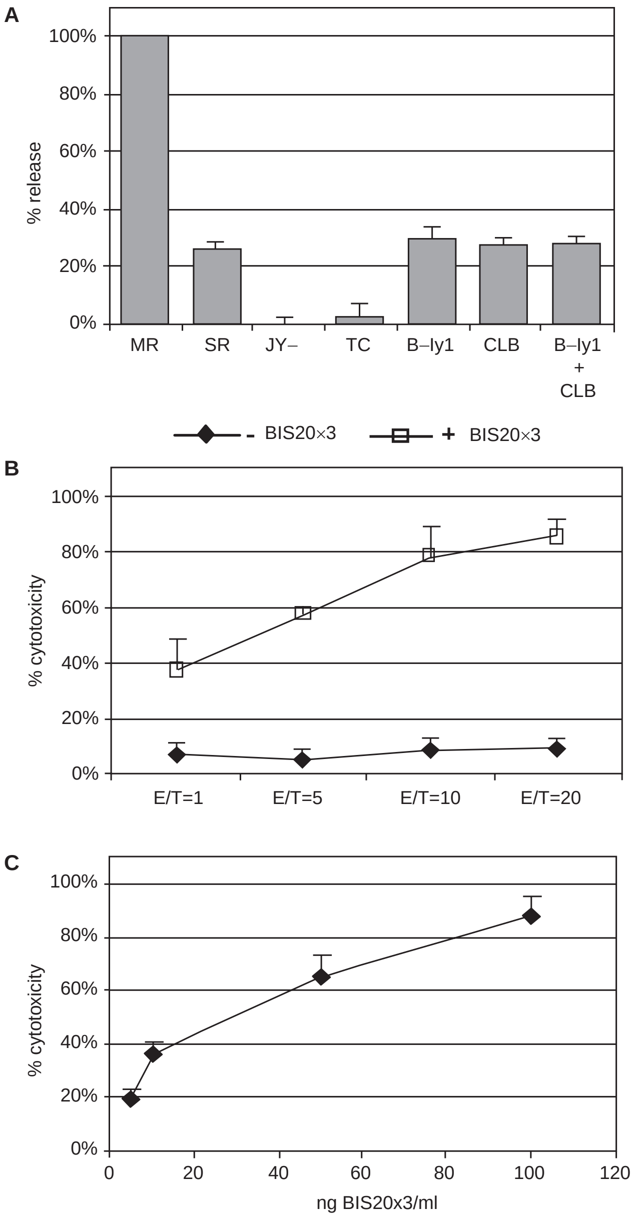

**Figure 4** In Figure 4A the calcein-release assay-controls are presented. The maximal release value (obtained from Triton-treated cells) was set to 100% and the following bars show the %-release related to this value. Shown are respectively: MR = maximal release, SR = spontaneous release, JY- = background fluorescence of medium in which unlabelled JY cells were present, TC = background fluorescence of medium in which unlabelled T-cells were present, B-ly1 = effect of 0.5 µg ml$^{-1}$ parental antibody B-ly1 on labelled JY cells, CLB = effect of 0.5 µg ml$^{-1}$ of other parental antibody CLB-T3/4.2B (0.5 µg ml$^{-1}$), and B-ly1 + CLB = addition of 0.5 µg ml$^{-1}$ B-ly1 + 0.5 µg ml$^{-1}$ CLB-T3/4.2B. The last 3 bars represent results obtained after adding the described antibodies to T- and B-cells at an E/T ratio of 20. Figure 4B shows representative results of the calcein-release assay. Cytotoxicity %s were calculated by implementing the formula described in the materials and methods section. BIS20x3 (0.5 µg ml$^{-1}$) was used to retarget PBL-derived T-cells to CD20-positive JY-cells at different E/T ratios. The results of Figure 4A and 4B were obtained in the same experiment. Figure 4C shows that the cytotoxicity is BIS20x3 concentration dependent (E/T-ratio of 20; 5, 10, 50 and 100 ng BIS20x3 ml$^{-1}$). The data presented in this figure could be reproduced in similar experiments background fluorescence. Addition of 1 µg ml$^{-1}$ B-ly1, CLB-T3/4.2B or a combination of both parental antibodies to wells containing T- and B-cells at an E/T-ratio of 20 had no effect. In Figure 4B a representative CTL-assay is described. It is shown that BIS20x3 re-targets PBL-derived, pre-activated T-cells to JY cells. With increasing effector/target cell ratios increased cytotoxicity %s were observed. These results could be reproduced readily indicating the good reproducibility of this non-radioactive cytotoxicity assay. Additionally, the results obtained by using the calcein release assay were essentially similar to results obtained by $^{51}$Cr-release (not shown). In none of the performed assays Rituximab was able to cause B-cell lysis in the calcein-release assay as it was performed here (essentially the results were similar to those obtained for B-ly1 described in Figure 4A). Finally, Figure 4C shows that the retargeting of T-cells to CD20 positive cells is BIS20x3-concentration-dependent and that cytotoxicity can be detected at BIS20x3 concentrations which are considerably lower (factor 50) than the anti-CD20-antibody concentrations needed to detect DNA fragmentation (Figure 2).

### DISCUSSION

The anti-CD20-antibody Rituximab is concerning its ability to induce tumour response rates the most effective anti-cancer antibody in the clinic at present. Its success is most probably due to a diversity of effects which are induced after binding to CD20. First, it has been shown that Rituximab can kill via complement mediated lysis. Very recently the C1q binding site on Rituximab has been identified (Idusogie et al, 2000). The second mechanism by which Rituximab is thought to elicit target cell death is via antibody-dependent cellular cytotoxicity. Although both mechanisms are potent cell death activating routes it is thought that the efficacy of Rituximab is at least partially due to additional mechanisms which are directly triggered by CD20 upon binding of the antibody. The function of CD20 as a signalling molecule has been investigated by several groups. It was demonstrated that upon binding of anti-CD20 antibodies 95% of all CD20 molecules present on the cell move to lipid rafts in the cell membrane which are known to be important in cellular signalling (Deans et al, 1998). It is thought that CD20 signals indirectly via tyrosine kinases which are known to be present in these rafts and which were shown to interact with CD20 (Deans et al, 1995) or directly by functioning as a $Ca^{2+}$-channel (Bubien et al, 1993; Tedder and Engel, 1994). Recently it was demonstrated that cross-linking of anti-CD20 antibodies on the surface of B-cells using F(ab′)2-fragments or Fc-receptor positive cells induces apoptosis in B-cells (Shan et al, 1998). The same group confirmed that protein tyrosine kinases, increased intracellular $Ca^{2+}$-concentrations, and caspases are involved (Shan et al, 2000). In the latter paper it was also shown that CD95 (*Fas*) becomes up-regulated after cross-linking of anti-CD20-antibodies on B-cells, but that the *Fas*-*Fas*L route is not important in the in vitro setting. However, this may have important consequences for the in vivo setting. Increased *Fas* expression could make Rituximab treated B-cells more sensitive for *Fas*L produced by other cells, especially by T-cells (Shresta et al, 1998). This is an important notion, especially in the context of T-cell re-targeting e.g. using the bi-specific antibody described in this paper.

Although several effector mechanisms seem to be triggered by Rituximab, 50% of NHL-patients do not respond. Recently, also patients with CD20-positive post-transplantation lymphoproliferative disorder (PTLD) were treated with Rituximab and also in this group 30% of the patients did not respond (Milpied et al, 2000). Various escape mechanisms which cause this non-responsiveness can be envisaged (e.g. a relative tumour cell-resistance to apoptosis induction). We hypothesize that the efficacy of treatment will increase when the delivered apoptotic signal can be strengthened and we think that this can be achieved by retargeting T-cells (professional killers) to CD20-positive cells using the bi-specific antibody BIS20x3 described in this paper.

To evaluate BIS20x3 it was investigated whether the purified product was able to induce the signals which are known to be relayed by each of its parental antibodies. Using the Burkitt lymphoma cell line Ramos as a model system in analogy to experiments performed by Shan et al (1998) we were able to demonstrate that apoptosis was induced after cross-linking of the parental anti-CD20 antibody B-ly1, Rituximab or BIS20x3. DNA fragmentation was detectable after overnight incubation using antibody concentrations of 0.1–0.5 $\mu g\ ml^{-1}$, which is considerably lower than the concentrations used by Shan et al (10 $\mu g\ ml^{-1}$). In the experiment depicted in Figure 2C it may seem that BIS20x3 cross-linking results in more efficient apoptosis induction (35% at a concentration of 5 $\mu g\ ml^{-1}$) than after cross-linking of B-ly1 or Rituximab (approximately 21% at a concentration of 5 $\mu g\ ml^{-1}$). However, it has to be pointed out that background apoptosis was rather high in this experiment compared to the experiments summarized in Figure 2A and 2B, which obviously influences the results. BIS20x3 induces simi percentages of apoptotic cells as B-ly1 or Rituximab, although BIS20x3 contains only 1 CD20-binding site while B-ly1 Rituximab contain two. It is likely that at the used B-ly1 and Rituximab concentrations these 2 antibodies bind monovalently. If Rituximab is cross-linked in vivo by Fc-receptor positive cells as was suggested by Shan, and if this phenomenon contributes significantly to Rituximab efficacy, our results imply that T-cell retargeting anti-CD20 bi-specific antibodies may be at least as effective in apoptosis induction when bound to B-cells as Rituximab.

The other chain of BIS20x3 targets CD3ε on T-cells. Binding of antibodies to CD3ε activates T-cells. We were able to demonstrate T-cell activation upon cross-linking of BIS20x3 on its surface using goat-anti-mouse F(ab′)2-fragments (Figure 3A). To obtain these results relatively high BIS20x3-concentrations have to be used (compare the amounts used in Figure 3A with the amounts used in to obtain the results presented in Figure 3B). T-cell activation could be enhanced dramatically by using Ramos cells as indirect cross-linkers instead of the F(ab′)2-fragments (Figure 3B). The enhanced T-cell activation found in this situation is most probably caused by co-stimulatory signals given by the Ramos cells. These results suggest that B-cell malignancies may be ideal targets for T-cell retargeting bi-specific antibody-based therapies because the retargeted T-cells will be activated most effectively (Chaperot et al, 2000). This finding also suggests that no additional co-stimuli need to be given, which was suggested when T-cells are retargeted to other types of malignant target cells (discussed in Kroesen et al, 1998). The results in Figure 3B also suggest that for optimal effects the bi-specific antibody concentration needs to be titrated, which may become an issue when dose-escalating clinical studies are planned. If the concentration of bi-specific antibody gets too high the binding sites on T- and B-cells may become saturated without cross-linking the cells to each other.

Although it is theoretically possible that our quadroma produces up to 10 different immunoglobulin molecules due to shuffling of the heavy and light chains (Kroesen et al, 1998), our purified product is very efficient in retargeting T-cells to B-cells (Figure 4). This was expected because in general the percentage of unwanted heavy/light chain combinations is low because each heavy chain has the highest affinity for its own light chain. Moreover, the most important and most abundant ‘contaminants’, the parental mono-specific species, were certainly not isolated (see Figure 1). The experiments above and the high retargeting efficiency therefore suggest that the bispecific antibody is present in abundant amounts. The high cytotoxicity observed may be due to optimal T-cell activation caused by co-stimulatory signals delivered by the B-cells and/or by the increased sensitivity of the B-cells for *Fas*L after binding of anti-CD20 antibodies. Although we did not address this question in this study it will be interesting to investigate if in the bi-specific setting, the apoptosis-inducing signal induced upon anti-CD20-crosslinking plays an important role or whether the T-cell-mediated signal is far more efficient. Another important question is if BIS20x3 is more efficient in treating lymphomas than CD19xCD3 bispecific antibodies. Several CD19xCD3 bispecific antibodies have been described recently (Daniel et al, 1998; Cochlovius et al, 2000; Löffler et al, 2000) in several antibody-formats (quadroma-derived, bi-specific single-chain and diabody respectively). The findings that CD20 seems to be higher expressed on B-cells compared to CD19 and that CD20 is not internalized after antibody binding in contrast to CD19-antibody complexes are often used as arguments for the use of CD20

*© 2001 Cancer Research Campaign*

antibodies (e.g. Press et al, 1989). In vivo comparison of CD3xCD19 antibodies with BIS20x3 would be very informative in this matter.

The application of T-cell retargeting anti-CD20 antibodies in therapies for B-cell malignancies may be an improvement upon Rituximab-based therapies. Our finding that T-cells are very efficiently activated in a setting in which B-cells are present is very promising in this respect. This characteristic is probably caused by the fact that unlike most other cancer cells, malignant B-cells may be capable to function as antigen-presenting cells (Chaperot et al, 2000). Also the recent finding that after cross-linking of anti-CD20-antibodies on B-cells *Fas* is up-regulated in these cells (Shan et al, 2000) could have major implications for T-cell retargeting-based lymphoma therapies, because this will make the B-cells even more sensitive for *Fas*L on activated T-cells.

### ACKNOWLEDGEMENTS

We would like to acknowledge G Mesander and H Moes for expert assistance during the FACS experiments. The help and advice of A Bakker, A Niemarkt, A ter Haar, J Dokter and E Bruin-van Dijk was appreciated during culturing, immunohistochemical assays and $^{51}$Cr-release assays. Y Groenewold isolated some of the bi-specific antibody. This work was funded by a grant of the European Community (BIO4-CT-97-2005).

### REFERENCES

Bubien JK, Zhou LJ, Bell PD, Frizzell RA and Tedder TF (1993) Transfection of the CD20 cell surface molecule in ectopic cell types generates Ca2+ conductance found constitutively in B lymphocytes. *J Cell Biol* **121**: 1121–1132

Chaperot L, Jacob MC, Molens JP, Manches O, Bensa JC and Plumas J (2000) From the study of tumor cell immunogenicity to the generation of antitumor cytotoxic cells in non-Hodgkin's lymphomas. *Leuk Lymphoma* **38**: 247–263

Cochlovius B, Kipriyanov SM, Stassar MJJG, Christ O, Schuhmacher J, Strauß G, Moldenhauer G and Little M (2000) Treatment of human B cell lymphoma xenografts with a CD3xCD19 diabody and T cells. *J Immunol* **165**: 888–895

Cragg MS, French RR and Glennie MJ (1999) Signalling antibodies in cancer therapy. *Curr Opin Immunol* **11**: 541–547

Daniel PT, Kroidl A, Kopp J, Sturm I, Moldenhauer G, Dörken B and Pezzutto A (1998) Immunotherapy of B-cell lymphoma with CD3x19 bispecific antibodies: Co-stimulation via CD28 prevents "veto" apoptosis of antibody-targeted cytotoxic T cells. *Blood* **92**: 4750–4757

Deans JP, Kalt L, Ledbetter JA, Schieven GL, Bolen JB and Johnson P (1995) Association of 75/80-kDa phosphoproteins and the tyrosine kinases Lyn, Fyn, and Lck with the B cell molecule CD20. *J Biol Chem* **270**: 22632–22638

Deans JP, Robbins SM, Polyak MJ and Savage JA (1998) Rapid distribution of CD20 to a low density detergent-insoluble membrane compartment. *J Biol Chem* **273**: 344–348

Helfrich W, Kroesen BJ, Roovers RC, Westers L, Molema G, Hoogenboom HR and De Leij L (1998) Construction and characterization of a bispecific diabody for retargeting T cells to human carcinomas. *Int J Cancer* **76**: 232–239

Heijnen IAFM and Van De Winkel JGJ (1997) Human IgG Fc receptors. *Int Rev Immunol* **16**: 29–56

Idusogie EE, Presta LG, Gazzano-Santoro H, Totpal K, Wong PY, Ultsch M, Meng YG and Mulkerrin MG (2000) Mapping of the C1q binding site on Rituxan, a chimeric antibody with a human IgG1 Fc. *J Immunol* **164**: 4178–4184

Kroesen BJ, Ter Haar A, Spakman H, Willemse P, Sleijfer DTh, De Vries EGE, Mulder NH, Berendsen HH, Limburg PC, the TH and De Leij L (1993) Local antitumour treatment in carcinoma patients with bi-specific-monoclonal-antibody-redirected T cells. *Cancer Immunol Immunother* **37**: 400–407

Kroesen BJ, Bakker A, Van Lier RAW, The HT and De Leij L (1995) Bispecific antibody mediated target-cell specific costimulation of resting T-cells via CD5 and CD28. *Cancer Res* **55**: 4409–4415

Kroesen BJ, Helfrich W, Molema G and De Leij L (1998) Bispecific antibodies for treatment of cancer in experimental animal models and man. *Adv Drug Deliv Rev* **31**: 105–129

Lichtenfels R, Biddison WE, Schulz H, Vogt AB and Martin R (1994) CARE-LASS (calcein-release-assay), an improved fluorescence-based test system to measure cytotoxic T lymphocyte activity. *J Immunol Meth* **172**: 227–239

Löffler A, Kufer P, Lutterbüse R, Zettl F, Daniel PT, Schwenkenbecher JM, Riethmüller G, Dörken B and Bargou RC (2000) A recombinant bispecific single-chain antibody, CD19xCD3, induces rapid and high lymphoma-directed cytotoxicity by unstimulated T lymphocytes. *Blood* **95**: 2098–2103

Milpied N, Vasseur B, Parquet N, Garnier JL, Antoine C, Quartier P, Carret AS, Bouscary D, Faye A, Bourbigot B, Reguerre Y, Stoppa AM, Bourquard P, Hurault de Ligny B, Dubief F, Mathieu-Boue A and LeBlond V (2000) Humanized anti-CD20 monoclonal antibody (Rituximab) in post transplant B-lymphoproliferative disorder: A retrospective analysis on 32 patients. *Ann Oncol* 11 (suppl1): S113–S116

Nicoletti I, Migliorati G, Pagliacci MC, Grignani F and Riccardi C (1991) A rapid and simple method for measuring thymocyte apoptosis by propidium iodide staining and flow cytometry. *J Immunol Meth* **139**: 271–279

Poppema S and Visser L (1987) Preparation and application of monoclonal antibodies: B cell panel and parafin tissue reactive panel. *Biotest Bull* **3**: 131–139

Press OW, Farr AG, Borroz KI, Anderson SK and Martin PJ (1989) Endocytosis and degradation of monoclonal antibodies targeting human B-cell malignancies. *Cancer Res* **49**: 4906–4912

Riethmuller G, Holz E, Schlimok G, Schmiegel W, Raab R, Hoffken K, Gruber R, Funke I, Pichlmaier H, Hirche H, Buggisch P, Witte J and Pichlmayr R (1998) Monoclonal antibody therapy for resected Dukes' C colorectal cancer: seven-year outcome of a multicenter randomized trial. *J Clin Oncol* **16**: 1788–1794

Rosen ST (Guest Ed.) (1999) In: Recent advances and future directions using monoclonal antibodies for B-cell malignancies. *Semin Oncol* **26** (suppl14): 2–122

Rudin CM and Thompson CD (1998) B-cell development and maturation. *Semin Oncol* **25**: 435–446

Shan D, Ledbetter JA and Press OW (1998) Apoptosis of malignant human B cells by ligation of CD20 with monoclonal antibodies. *Blood* **91**: 1644–1652

Shan D, Ledbetter JA and Press OW (2000) Signaling events involved in anti-CD20-induced apoptosis of malignant human B cells. *Cancer Immunol Immunother* **48**: 673–683

Shresta S, Pham CTN, Thomas DA, Graubert TA and Ley TJ (1998) How do cytotoxic lymphocytes kill their targets? *Curr Opin Immunol* **10**: 581–587

Tax WJM, Willems HW, Reekers PPM, Capel PJ and Koene RAP (1983) Polymorphism in mitogenic effect of IgG1 monoclonal antibodies against T3 antigen on human T-cells. *Nature* **304**: 445–447

Tedder TF and Engel P (1994) CD20: A regulator of cell cycle progression of B-lymphocytes. *Immunol Today* **15**: 450–454

Van derBruggen P, Bastin J, Gajewski T, Coulie P, Boel P, De Smet C, Traversers C, Townsend A and Boon T (1994) A peptide encoded by human gene MAGE-3 and presented by HLA-A2 induces cytolytic T lymphocytes that recognize tumor cells expressing MAGE-3. *Eur J Immunol* **24**: 3038–3043

Van Lier RA, Boot JH, De Groot ER and Aarden LA (1987a) Induction of T cell proliferation with anti-CD3 switch-variant monoclonal antibodies: effects of heavy chain isotype in monocyte-dependent systems. *Eur J Immunol* **17**: 1599–1604

Van Lier RA, Boot JH, Verhoeven AJ, De Groot ER, Brouwer M and Aarden LA (1987b) Functional studies with anti-CD3 heavy chain isotype switch-variant monoclonal antibodies. Accessory cell-independent induction of interleukin 2 responsiveness in T-cells by epsilon-anti-CD3. *J Immunol* **139**: 2873–2879

© 2001 Cancer Research Campaign 
